# Incapacitating pain from Tenofovir Induced Hypophosphatemic Osteomalacia in a Hemophilia Patient – A Case Report

**DOI:** 10.1080/24740527.2020.1838890

**Published:** 2020-12-14

**Authors:** Emma Woo, Dinesh Kumbhare, Paul Winston

**Affiliations:** a Faculty of Medicine, University of British Columbia, Vancouver, British Columbia, Canada; b Department of Medicine, Division of Physical Medicine and Rehabilitation, University of Toronto, Toronto, Ontario, Canada

**Keywords:** osteomalacia, Fanconi syndrome, hemophilia, human immunodeficiency virus

## Abstract

**Background:** Pain in patients with hemophilia is common and usually a result of arthropathy. Clinicians should, however, consider a wide range of etiologies for pain in patients with hemophilia including infection, osteoporotic fractures, arthritis, and osteomalacia.

**Aims:** This case demonstrates an instance of poorly localized back and hip pain, severe enough to prevent ambulation, caused by hypophosphatemic osteomalacia due to tenofovir treatment for blood transfusion acquired Human Immunodeficiency Virus (HIV) in a patient with hemophilia A.

**Methods:** Case Report.

**Results:** With termination of tenofovir treatment, this patient returned to baseline function.

**Conclusion:** This report serves to emphasize the need for accurate diagnosis of pain in hemophilia patients, especially among the aging demographic of people with hemophilia in which there is a significant likelihood of an HIV infection and among patients who may be on Pre-exposure Prophylaxis (PrEP) or clinical trials involving tenofovir.

## RÉSUMÉ

**Contexte**: La douleur chez les patients hémophiles est courante et résulte généralement d’une arthropathie. Les cliniciens doivent cependant envisager un large éventail d’étiologies pour la douleur chez les patients hémophiles y compris l’infection, les fractures ostéoporotiques, l’arthrite et l’ostéomalacie.

**Buts**: Ce cas montre un exemple de douleur mal localisée au dos et à la hanche, suffisamment grave pour empêcher la marche, provoquée par une ostéomalacie hypophosphatémique attribuable au traitement par ténofovir pour le virus de l’immunodéficience humaine (VIH) acquis par transfusion sanguine chez un patient atteint d’hémophilie A.

**Méthodes**: Rapport de cas.

**Résultats**: Avec l’arrêt du traitement par ténofovir, ce patient a récupéré sa capacité fonctionnelle de départ.

**Conclusion**: Ce rapport souligne la nécessité d’un diagnostic précis de la douleur chez les patients hémophiles, en particulier parmi la population vieillissante des personnes atteintes d’hémophilie chez qui il existe une forte probabilité d’infection au VIH et parmi les patients auxquels la prophylaxie pré-exposition (PrEP)est administrée ou qui participent à des essais cliniques impliquant le ténofovir.

## Introduction

Pain is commonplace in patients with hemophilia and often arises as the result of hemarthrosis, joint bleeds that may cause progressive joint damage and have a significant effect on quality of life and mobility.^1^ However, it cannot be assumed that severe pain is due to hemarthrosis-related joint damage. Clinicians should consider a wide range of etiologies for pain in patients with hemophilia. Acute causes include infection, osteoporotic fractures, bleeds, with chronic causes including arthritis, and osteomalacia. A comprehensive medical history may reveal unanticipated causes, including side effects from medications such as tenofovir, specifically tenofovir disoproxil fumarate. This case demonstrates a rare instance of poorly localized back and hip pain, severe enough to prevent ambulation in a patient with hemophilia A caused by hypophosphatemic osteomalacia due to tenofovir treatment for blood transfusion–acquired human immunodeficiency virus (HIV). In the early 1980s, before blood products were tested or heated, nearly 50% of the U.S. population with hemophilia had become infected with HIV from blood transfusions.^2^ Among the aging population of people with hemophilia, there is a significant likelihood of an HIV infection.^2^ Other uses of tenofovir include pre-exposure prophylaxis (PrEP) and treatment of chronic hepatitis B infections.

## Case Report

This study conforms to all CARE guidelines and reports the required information accordingly (see Supplementary Checklist). A 44-year-old man with hemophilia A with a known history of polyarticular hemarthrosis presented with worsening back and hip pain over the course of several months. His pain was diffuse, more prominent on the right than on the left, and radiated into his thighs. He was now ambulating using a left-sided crutch, with an antalgic gait, complaining of chronic headaches and neck pain. He had been assessed by internal medicine, physiatry, and neurology and no cause for his pain was found. His ambulation had deteriorated to requiring a wheelchair for mobility due to severe widespread pain and he was receiving physiotherapy. Review of systems was significant for decreased energy and pain in ribs when coughing.

**Figure 1.**
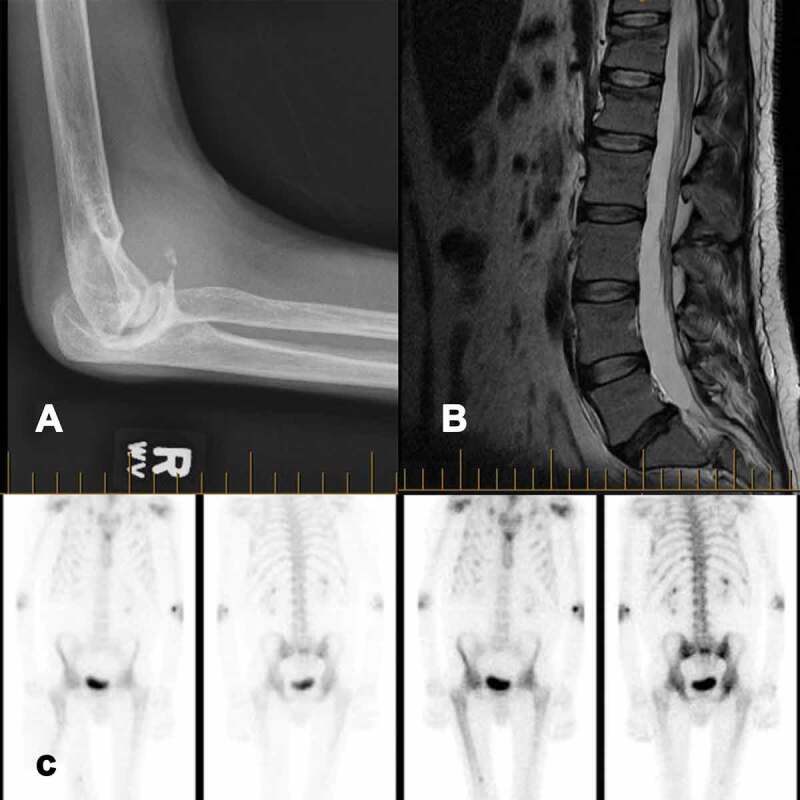
(a) x-ray of right elbow showing significant joint degeneration. (b) MRI of the spine showing superior end plate compression fractures in T12 and L1 with minimal loss of height. (c) Bone scan revealing widespread increased uptake

Relevant medical history included hepatitis C and HIV, both acquired due to transfusion of contaminated blood products. His hepatitis C was previously treated with pegylated interferon and ribavirin. HIV was suppressed with highly active antiretroviral therapy, which included Truvada (emtricitabine/tenofovir). His viral loads were consistently well controlled with less than 40 copies/mL. The patient’s hemophilia was being treated with factor VIII daily prophylaxis because of a high rate of bleeds, with 10 bleeds in the previous year. The patient suffered from extensive multijoint hemarthrosis and degeneration (see Figure 1a for example of elbow degeneration), resulting in two right knee arthroplasties, a left knee arthroplasty, and a right ankle calcaneal osteotomy.

His treatment and workup began with a prescription of oxycodone 2 mg tabs 1 to 2 q4h, and the early diagnostics focused on the hip with an early tentative diagnosis of right hip osteoarthritis. Investigations performed included an x-ray of the lumbar spine and hips followed by computed tomography of the lower lumbar spine and pelvis, which showed no evidence of hemarthrosis, hematoma, or other acute pathology that could explain the pain. Avascular necrosis and osteochondritis dissecans had been ruled out on magnetic resonance imaging (MRI; see Figure 1b). A bone scan revealed moderate uptake in both hips and mid-femurs, previous multiple rib fractures, and a possible old left femur fracture and mild uptake in the spine, shoulders, hips, and elbows consistent with arthropathy (see Figure 1c).

On physical exam, he displayed thoracic kyphosis, enlarged elbow bony prominences bilaterally, and atrophied upper body musculature. His back and hip pain were indescribable and now so severe that a supine examination could not be performed. He had swelling over the upper sacral area with no acute local or spinal tenderness.

The patient underwent a nerve conduction study, which revealed small amplitude tibial and peroneal motor responses as well as absent sural nerve response indicating a mild peripheral neuropathy. His bone density studies revealed osteoporosis in the pelvic girdle with a T-score as low as −4.4 in the femoral neck. The patient had normal electrolyte levels in his blood work, including phosphate and calcium levels, a normal glomerular filtration rate, and an elevated alkaline phosphatase. He had a normal C-reactive protein, creatinine kinase, and serum protein electrophoresis.

His pain persisted over months, and a subsequently performed noncontrast lumbar MRI demonstrated superior end plate compression fractures in T12 and L1 with minimal loss of height that had not previously been reported on x-ray (see Figure 1b). One year after initial presentation, an endocrinologist noted that the severe pain, in the context of glycosuria, a protein-to-creatinine ratio of 270 g/mol, hypokalemia, hypophosphatemia (0.54–0.63 mmol/L), elevated urine phosphate of 32 mmol/day, and elevated alkaline phosphatase (ALP) could be due to malabsorption in the proximal renal tubule and be indicative of Fanconi syndrome. Given that tenofovir has a rare but known association with Fanconi syndrome, which in turn can develop into hypophosphatemic osteomalacia causing severe pain, he was advised to stop taking tenofovir and begin taking phosphate, vitamin D, and calcium. After discontinuation of tenofovir, the patient continued on his four other HIV medications and showed rapid improvement over the following months. He could walk without assistance for over 5 min, had returned to work, and no longer experienced severe headaches.

## Discussion

Pain is commonplace within the hemophiliac population.^1,3^ This case demonstrates a patient with longstanding hemophilia and several previous bony complications who presented with severe pain and no clearly defined etiology. For our case, the etiology of pain was difficult to determine. Initial history, physical exam, and imaging were nondiagnostic, and the eventual diagnosis of Fanconi syndrome was made based on increased urinary protein, urinary glucose, elevated ALP.

There is a growing database of literature on the assessment and management of pain in hemophilia.^3,4^ This literature, however, primarily addresses acute and chronic pain as it relates to arthropathy from hemarthrosis and does not specifically address the need to accurately diagnose the pathology of the underlying pain. Though the differential diagnosis for pain in hemophilia is usually limited to acute pain from arthropathies, there are other pathological entities that account for pain in people with hemophilia (see Table 1). Downstream effects of hemarthrosis and hematomas can also lead to chronic pain from synovitis, degenerative arthropathy, and pseudotumors.^5,6^ Hemophilia pain can also have extrinsic associations, including deconditioning, associated illnesses, neurological injuries/impairments, medication side effects, and trauma. Determining the characteristics of the pain is important in distinguishing arthropathies from other causes. Specifically, pain with arthropathies tends to be initially acute, localized, and severe and become “arthritic” with time and increased joint damage.^4^ Bone pain, on the other hand, will typically present as a dull aching pain unless due to a fracture, which will cause sharp, radiating pain.^7^

**Table 1.** Etiologies of pain in patients with Hemophilia

Etiology	Location of Pain	Relevant papers
Hemophilic Arthropathy	Knee Hip Elbow Ankle Wrist	^5, 17-19^
Acute Hemarthrosis	Knee Hip Ankle Wrist	
Hematoma	Psoas muscle Hip Lumbar Spine	^20, 21^
Synovitis	Knee Ankle Wrist	^22, 23^
Pseudotumor	Hip Lumbar Spine Wrist Abdomen Cranium	^24, 25^
Peripheral Nerve complications (Including HIV neuropathy)	Femoral Sciatic Peroneal Distal extremities	^26^
Osteoporotic fractures	Spine Wrist Hips Shoulder	^6, 27^

In patients with hemophilia who would have received blood transfusions in the 1980s, it is important to consider the possibility of an infusion-acquired HIV infection that is being treated with antiretrovirals with pain-causing side effects.^6^ There is a known association between Fanconi syndrome and resulting osteomalacia in tenofovir-treated patients.^8–10^ Fanconi syndrome is a rare disorder resulting in excess excretion of glucose, bicarbonate, phosphates, uric acid, potassium, and some amino acids. Patients usually present with normal glomerular function, pain, muscle weakness, and decreased energy.^11^ Fanconi syndrome is defined as inadequate reabsorption in the proximal renal tubules of the kidney and can be caused by a range of underlying congenital or acquired diseases, as well as drug toxicity. A review of the U.S. Food and Drug Administration’s Adverse Event Reporting System from 2001 to 2006 identified 164 tenofovir fumarate–treated patients with HIV with Fanconi syndrome.^8^ Patients with Fanconi syndrome subsequently have risks of electrolyte abnormalities and acid-base disturbances and are at risk for hypophosphatemic osteomalacia.^12^ In turn, these patients have an increased burden of pain, increased fracture risk, and decreased quality of life. Given the potential for diagnostic ambiguity, these patients are at risk for delayed diagnosis. Treatment of Fanconi syndrome and complications of hypophosphatemic osteomalacia involves treating the cause and generally results in a good prognosis.^12,13^

In patients with confirmed HIV who are taking antiretrovirals or those using PrEP, the National Institute of Health guidelines recommend that basic chemistry be done every 6 months and urinalysis be done every 12 months. For patients specifically on tenofovir, it is recommended that both basic chemistry and urinalysis be done every 6 months.^14^ Although ALP is not specifically referenced in these guidelines, alanine aminotransferase, aspartate aminotransferase, and bilirubin are recommended every 6 months and it would be reasonable to add ALP to this list.

Tenofovir was being used in this case as HIV treatment, but it can also be used to treat hepatitis B or prevent HIV. As treatment or PrEP for HIV it is usually combined with emtricitabine at a dose around 300 mg/200 mg. Though it has a relatively good safety profile, there are numerous described cases of nephrotoxicity,^9,10,15^ the development of which should be carefully monitored. Though tenofovir disoproxil fumarate is the typical drug used for HIV prophylaxis, cases have been reported of a prodrug (tenofovir adafamide) having lesser effects on kidney function.^15^

This is especially important given that the SARS-CoV-2 (COVID-19) epidemic has sparked a renewed interest in antiviral medications. A randomized controlled trial is underway testing the efficacy of tenofovir disoproxil/emtricitabine as a COVID-19 prophylaxis for health care professionals.^16^ There should be significant consideration for the potential side effects that participants may face, including rare side effects such as Fanconi syndrome. Practitioners should also recognize that PrEP is typically distributed independently and confidentially from specialized clinics, so whether or not someone is on PrEP may not be readily available based on pharmacy or referring physician records.

In patients presenting with pain unexplained after performing the usual history, physical exam, and imaging findings, one should consider osteomalacia secondary to tenofovir-induced Fanconi syndrome. Early evaluation for Fanconi syndrome and subsequent osteomalacia can involve using accredited pain assessment tools, bone mineral density studies, and monitoring of kidney function, including following electrolytes, urinary protein, urinary glucose, and plasma/urinary phosphate levels.

## Conclusions

This case reveals the importance of keeping a wide differential open for patients with hemophilia experiencing severe pain, particularly those experiencing widespread multijoint pain. The approach should begin with a thorough history including an extensive pain and medication history. Given the prevalence of contaminated blood transfusion in the 1980s, it is important to specifically discuss bloodborne illness, the treatments that are being used to combat it, and their potential side effects.

This case also demonstrates the importance of being able to characterize pain in a patient with hemophilia in order to appropriately determine the etiology of the pain and appropriate treatment. In addition to new assessment tools to describe, monitor, and guide pain treatment for people with hemophilia, increased focus should be made on diagnosing the etiology of the pain itself.

## Supplementary Material

Supplemental Material
