# Supplementary material for: Incapacitating pain from Tenofovir Induced Hypophosphatemic Osteomalacia in a Hemophilia Patient – A Case Report
Source: Can J Pain. 2020 Dec 14;4(1):287–91. doi: 10.1080/24740527.2020.1838890 (PMC7951154; doi:10.1080/24740527.2020.1838890)
Supplement: Supplemental Material [file UCJP_A_1838890_SM8486.pdf]

CARE case report guidelines

CARE Checklist of information to include when writing a case report

(cc) BY-NC-ND

Topic	Item	Checklist item description	Reported on Line
Title	1	The diagnosis or intervention of primary focus followed by the words "case report"	1-2
Key Words	2	2 to 5 key words that identify diagnoses or interventions in this case report, including "case report"	63
Abstract (no references)	3a	Introduction: What is unique about this case and what does it add to the scientific literature?	81-84
	3b	Main symptoms and/or important clinical findings	81-84
	3c	The main diagnoses, therapeutic interventions, and outcomes	75-78
	3d	Conclusion—What is the main "take-away" lesson(s) from this case?	78-81
Introduction	4	One or two paragraphs summarizing why this case is unique (**may include references**)	91-108
Patient Information	5a	De-identified patient specific information.	92-94
	5b	Primary concerns and symptoms of the patient.	92-108
	5c	Medical, family, and psycho-social history including relevant genetic information	101-108
	5d	Relevant past interventions with outcomes	110-118
Clinical Findings	6	Describe significant physical examination (PE) and important clinical findings.	120-131
Timeline	7	Historical and current information from this episode of care organized as a timeline	133-143
Diagnostic Assessment	8a	Diagnostic testing (such as PE, laboratory testing, imaging, surveys).	133-143
	8b	Diagnostic challenges (such as access to testing, financial, or cultural)	133-143
	8c	Diagnosis (including other diagnoses considered)	133-143
	8d	Prognosis (such as staging in oncology) where applicable	n/a
Therapeutic Intervention	9a	Types of therapeutic intervention (such as pharmacologic, surgical, preventive, self-care)	110-143
	9b	Administration of therapeutic intervention (such as dosage, strength, duration)	110-111
	9c	Changes in therapeutic intervention (with rationale)	110-143
Follow-up and Outcomes	10a	Clinician and patient-assessed outcomes (if available)	110-143
	10b	Important follow-up diagnostic and other test results	110-143
	10c	Intervention adherence and tolerability (How was this assessed?)	110-143
	10d	Adverse and unanticipated events	n/a
Discussion	11a	A scientific discussion of the strengths AND limitations associated with this case report	146-150
	11b	Discussion of the relevant medical literature **with references**.	146-204
	11c	The scientific rationale for any conclusions (including assessment of possible causes)	207-217
	11d	The primary "take-away" lessons of this case report (without references) in a one paragraph conclusion	214-217
Patient Perspective	12	The patient should share their perspective in one to two paragraphs on the treatment(s) they received	n/a
Informed Consent	13	Did the patient give informed consent? Please provide if requested	Yes ☒ No ☐
